# PangyPlot: multi-scale interactive visualization of pangenome variation graphs

**DOI:** 10.1093/bioinformatics/btag619

**Published:** 2026-08-18

**Authors:** Scott Mastromatteo, Shalvi Chirmade, Delnaz Roshandel, Bhooma Thiruvahindrapuram, Zhuozhi Wang, Rohan V Patel, Wilson W L Sung, Amirhossein Hajianpour, Cheng Wang, Fan Lin, Katherine Keenan, Julie Avolio, Paul D W Eckford, Felix Ratjen, Canadian Cystic Fibrosis Gene Modifier Consortium, Lisa J Strug, Katherine Keenan, Felix Ratjen, Johanna Rommens, Melinda Solomon, Candice Bjornson, Mark Chilvers, April Price, Michael Parkins, Michael Derynck, Emmanuelle Brochiero, Lara Bilodeau, Dimas Mateos-Corral, Daniel Hughes, Mary Jane Smith, Nancy Morrison, Janna Brusky, Anne Stephenson, Elizabeth Tullis, Bradey Quon, Jaled Yehya, Winnie M Leung, Andre Cantin, Larry Lands, Lisa J. Strug

**Affiliations:** Program in Genetics and Genome Biology, The Hospital for Sick Children, Toronto, ON, M5G 0A4, Canada; The Centre for Applied Genomics, The Hospital for Sick Children, Toronto, ON, M5G 0A4, Canada; Program in Genetics and Genome Biology, The Hospital for Sick Children, Toronto, ON, M5G 0A4, Canada; The Centre for Applied Genomics, The Hospital for Sick Children, Toronto, ON, M5G 0A4, Canada; Program in Genetics and Genome Biology, The Hospital for Sick Children, Toronto, ON, M5G 0A4, Canada; The Centre for Applied Genomics, The Hospital for Sick Children, Toronto, ON, M5G 0A4, Canada; The Centre for Applied Genomics, The Hospital for Sick Children, Toronto, ON, M5G 0A4, Canada; The Centre for Applied Genomics, The Hospital for Sick Children, Toronto, ON, M5G 0A4, Canada; The Centre for Applied Genomics, The Hospital for Sick Children, Toronto, ON, M5G 0A4, Canada; Department of Pediatric Laboratory Medicine, The Hospital for Sick Children, Toronto, ON, M5G 1H3, Canada; The Centre for Applied Genomics, The Hospital for Sick Children, Toronto, ON, M5G 0A4, Canada; Program in Genetics and Genome Biology, The Hospital for Sick Children, Toronto, ON, M5G 0A4, Canada; Program in Genetics and Genome Biology, The Hospital for Sick Children, Toronto, ON, M5G 0A4, Canada; Program in Genetics and Genome Biology, The Hospital for Sick Children, Toronto, ON, M5G 0A4, Canada; Translational Medicine, The Hospital for Sick Children, Toronto, ON, M5G 0A4, Canada; Translational Medicine, The Hospital for Sick Children, Toronto, ON, M5G 0A4, Canada; Mission Programs, Cystic Fibrosis Canada, Toronto, ON, M4R 1K8, Canada; Translational Medicine, The Hospital for Sick Children, Toronto, ON, M5G 0A4, Canada; Division of Respiratory Medicine, The Hospital for Sick Children, Toronto, ON, M5G 1X8, Canada; Program in Genetics and Genome Biology, The Hospital for Sick Children, Toronto, ON, M5G 0A4, Canada; The Centre for Applied Genomics, The Hospital for Sick Children, Toronto, ON, M5G 0A4, Canada; Departments of Statistical Sciences and Computer Science, University of Toronto, Toronto, ON, M5G 1X6, Canada; Biostatistics Division Dalla Lana School of Public Health, University of Toronto, Toronto, ON, M5S 3E3, Canada

## Abstract

**Summary:**

Pangenome variation graphs integrate multiple samples into a unified representation, mitigating the reference bias inherent to linear genomes. However, these graphs can be large and structurally complex. Existing visualization tools are each confined to a fixed scale of resolution, requiring researchers to switch between multiple tools to examine variation at different levels of detail. PangyPlot is an interactive pangenome browser designed for multi-scale exploration of reference variation graphs from full chromosome to nucleotide-level sequence segments. PangyPlot anchors navigation to linear reference coordinates, organizes variation into hierarchical bubble structures, and uses a force-directed layout engine for automatic node arrangement.

**Availability and implementation:**

An instance preloaded with data is available at https://pangyplot.research.sickkids.ca. Source code and documentation are openly available at https://github.com/strug-hub/pangyplot under the MIT License.

## 1 Introduction

Linear reference genomes provide a shared coordinate system for genomic research and collaboration. However, they are susceptible to reference bias: systematic underrepresentation of variants proportional to their divergence from the reference (Brandt *et al.* 2015). This bias disproportionately affects populations that are genetically dissimilar to the reference, potentially perpetuating disparities in genetic research. Pangenome graphs have been proposed as an alternative (Computational Pan-Genomics Consortium 2016), combining *de novo* assemblies from multiple individuals into a unified representation of observed variation. The Human Pangenome Reference Consortium (HPRC) (Liao *et al.* 2023) has produced graph-based references using this approach. Pangenomes employ a variation graph representation where DNA sequences are nodes and variation is encoded in branching edges that form bubbles (also called snarls). A bubble is a subgraph with a single source and sink node that can be nested or form higher-order structures such as bubble chains; chains are formed when adjacent bubbles share source and sink nodes (Fig. S1) (Paten *et al.* 2018). As these topological features encode the variation represented in pangenomes, producing interpretable visualizations is essential for pangenome-based genomic research.

Eukaryotic-scale pangenome visualization remains difficult, owing to the size of variation graph data and the challenge of representing both structural variants (SVs) and single nucleotide variants (SNVs). The most widely used sequence graph visualization tools each operate at a fixed level of resolution: odgi draw (Guarracino *et al.* 2022) is helpful as it can produce a static image at a chromosomal scale but it is not ideal for small variant visualization nor does it operate interactively; Bandage (Wick *et al.* 2015) loads the entire graph into memory, enabling investigation of pre-extracted subregions; and Sequence Tube Map (Beyer *et al.* 2019) renders haplotype paths at the SNV level but becomes difficult to interpret as graph complexity increases. Currently these tools can be used in combination to navigate the full range of genomic scale, although this can be cumbersome requiring format conversions, subgraph extraction, and manual effort at each step. To address the need for a comprehensive pangenome browser, we present PangyPlot, which enables interactive exploration of variation graphs from the chromosomal scale to single nucleotide resolution. PangyPlot front-loads a higher setup cost to enable low-latency queries, making it best suited to pangenomes that are indexed once and queried many times through a shared web service.

## 2 Implementation

**Figure 1:**
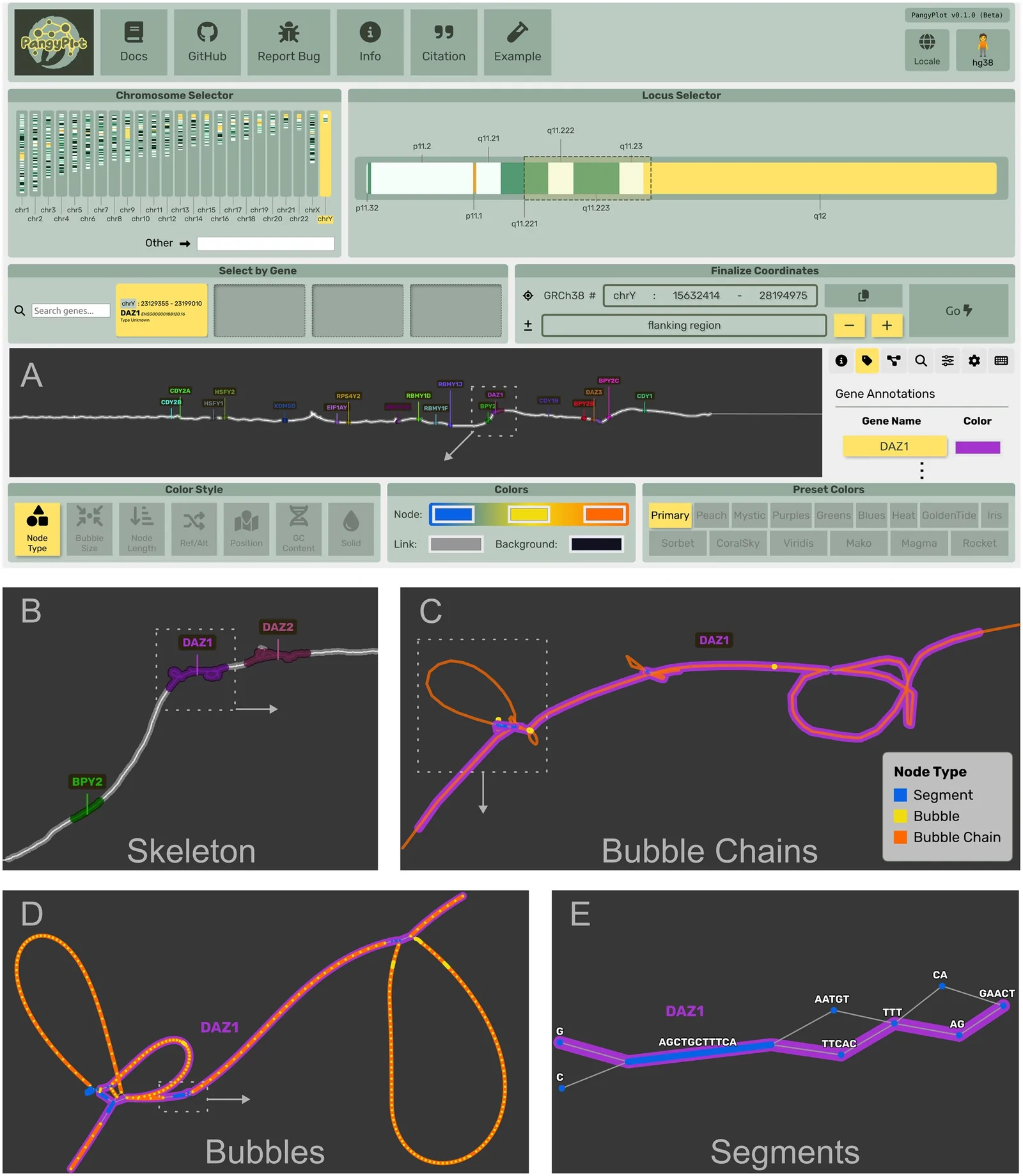
PangyPlot interface and multi-resolution pangenome visualization. (A) A large region of chromosome Y shown in the PangyPlot browser. The interface provides chromosome selection with ideogram, gene search, coordinate entry, interactive graph canvas, and customizable color palettes. (B–E) Illustrate multi-resolution navigation from chromosome-scale overview to nucleotide detail (dotted boxes). (B) Skeleton view: a simplified representation of the graph topology. (C) Bubble chain view: the *DAZ1* locus with bubble chains (orange) taking visual priority. (D) Bubble view: all bubbles within a chain (yellow) are visible and can be popped to reveal contents. (E) Segment view: bubble popping exposes individual sequence segments (blue) with base-pair-level resolution.

PangyPlot is a Python-based web application that can be hosted locally or prepared as a public server (Fig. S2). Users access an interface to explore a preloaded variation graph at multiple scales (Fig. 1). The design centres on three core principles that are explained in this section.

### 2.1 Reliance on a primary reference

PangyPlot requires a GFA file and a primary reference path name, gene annotations in GFF3 format are optional. Cytoband files for rendering chromosome ideograms are optional; several model organisms are preloaded and pseudo-cytobands can be generated for non-model organisms. Segments, links, and paths within the GFA are parsed and indexed in SQLite databases with complementary in-memory indexes (see Supplementary Methods 1 for details). Segments along the reference path are assigned linear reference coordinates. This anchors the graph to a familiar coordinate system, enabling region-based querying with standard genomic intervals and simplifying integration of gene annotations.

### 2.2 Bubble-aware visualization

Fully rendering all links and segments of a large GFA interactively is infeasible. Instead, PangyPlot uses BubbleGun (Dabbaghie *et al.* 2022) to calculate bubbles and bubble chains, organizing variation into hierarchical structures. When zoomed out, only chains and bubbles are rendered as circles drawn on simplified polylines. Individual segments within a bubble can be revealed by interactively popping (expanding) it (Fig. S3). This both reduces the number of nodes drawn, improving rendering performance, and manages visual complexity by presenting detail only on demand. Because complexity is bounded in this way, a complete chromosome remains explorable without extracting a subregion in advance.

### 2.3 Force-directed graph layout

PangyPlot relies on an initial two-dimensional layout generated by odgi layout (Heumos *et al.* 2024), the only method feasible at chromosome scale; recent GPU acceleration (Li *et al.* 2024) has reduced computation to minutes per chromosome. The layout assigns coordinates to each GFA segment, from which a skeleton graph structure is built using grid-based spatial simplification. When zoomed out, the skeleton is drawn as simple polylines. When zoomed in sufficiently, graph structures (chains, bubbles, and segments) are rendered directly using the D3 force-directed graph engine. Nodes and links are rendered to convey relative size differences of the graph components (Fig. S4). Custom forces dynamically arrange elements in real time to reduce the need for manual repositioning. These include a layout force that pulls nodes toward their initial layout coordinates, circular forces that shape bubbles around their source and sink, and a bubble-pop force that smoothly expands bubble contents (Fig. S5).

## 3 Application

### 3.1 Preprocessing performance

To evaluate feasibility of PangyPlot on eukaryotic data, we indexed both the PGGB (Garrison *et al.* 2024) and Minigraph-Cactus (Hickey *et al.* 2024) builds of the HPRC Release 1 human pangenome on a laptop (2.1 GHz Intel Core i5, 16 GB RAM). Timings are listed in Table S1. For the average autosome, PGGB chromosomes took 9.1 minutes and Minigraph-Cactus (GRCh38 reference) took 5.9 minutes of ingestion time into PangyPlot. Memory peaked below 13 GB across all chromosomes. Indexing is a one-time cost: once complete, the resulting indexes support rapid interactive queries that can also be deployed as a public server. Notably, for smaller GFA files typical of what Bandage can render, indexing completes in seconds. After indexing, the full Minigraph-Cactus pangenome database occupies 38 GB on disk. At runtime, indexes load in under 10 seconds and use 275 MB of RAM, making it practical to serve even large pangenomes from modest hardware. As a scale test, we also indexed the unreleased HPRC Release 2 Minigraph-Cactus graph, which contains roughly 5× more samples and averaged 12.7 minutes per autosome (Table S2).

### 3.2 Case study: Chromosome 7

To demonstrate PangyPlot’s functionality and compare it to existing tools, we constructed a Minigraph-Cactus chromosome 7 variation graph from PacBio long-read assemblies of individuals (*n* = 101, Supplementary Methods 2) participating in the Canadian Cystic Fibrosis (CF) Gene Modifier Study. Chromosome-scale visualization was achieved in PangyPlot following the odgi layout (Fig. S6), while full chromosome rendering is not feasible using Bandage or Sequence Tube Map.

We examined a 12 kb region encompassing *CFTR* exons 10 and 11 and an upstream polyT repeat that can alter splicing (Fig. S7a–e). This region contains the most common CF-causing variant (p.Phe508del), consistently observed in *cis* with the (TG)_10_T_9_ allele, shown with an example using the PangyPlot haplotype path highlighting feature. PangyPlot and Bandage produced comparable renderings, however additional effort is required to annotate and position nodes in Bandage. Sequence Tube Map also showed haplotype paths but was difficult to interpret in complex regions (Fig. S8). The odgi draw command could not produce effective visuals at the small variant level (Fig. S9).

We also examined the chromosome 7 *PRSS1*-*PRSS2* trypsinogen locus, which harbours a common 10 kb copy number variant (CNV) associated with intestinal obstruction at birth in CF (Mastromatteo *et al.* 2023). Most individuals carry either three or five copies with high sequence similarity (>90%). Both GRCh38 and CHM13 represent only the 3-copy allele, and PacBio HiFi read alignments failed to align 5-copy carriers due to reference bias (Fig. S10). In PangyPlot, the CNV was represented as a large bubble that could be expanded to reveal the full five-copy haplotype structure, with an animated path highlighting enabling per-sample allele visualization (Fig. S7f–i). Bandage and odgi draw produced comparable structures but required subsetting of the graph file and manual annotation of the genes in Bandage.

### 3.3 Conclusion

Table S3 shows a breakdown comparing the different visualization tools, and Table S4 provides a comprehensive list of features in PangyPlot. We recommend Sequence Tube Map for studying small-variant haplotype distribution, odgi draw to rapidly view large-scale structure, and Bandage as a mature, lightweight option for small GFA files. What separates PangyPlot from existing tools is the ability to span this full range: to render a static full-chromosome view in addition to individual segments and links, seamlessly and without requiring graph subsetting. PangyPlot is best-suited when pangenome visualization must be accessed repeatedly, across scales or by many users.

## Supplementary Material

btag619_Supplementary_Data

## Data Availability

The data underlying this article are available in Zenodo at https://doi.org/10.5281/zenodo.15311998, https://doi.org/10.5281/zenodo.19580038, and https://doi.org/10.5281/zenodo.17174108.
