# Supplementary material for: PangyPlot: multi-scale interactive visualization of pangenome variation graphs
Source: Bioinformatics. 2026 Aug 18;42(8):btag619. doi: 10.1093/bioinformatics/btag619 (PMC13523859; doi:10.1093/bioinformatics/btag619)
Supplement: Supplementary file 1 [file btag619_supplementary_data.docx]

Supplementary Material for

PangyPlot: multi-scale interactive visualization of pangenome variation graphs

# Supplementary Figures

**Figure S1.** Modeling genetic variation using graph-based structures. **a**, Linear reference sequence with example haplotypes aligned. Differences relative to the reference are highlighted in red. **b**, Graph-based representation of the haplotypes. Each of the four biallelic variants is represented as a bubble: a source node with bifurcating edges to two allele nodes and converging on a single sink node. **c**, A specific haplotype can be defined as a path through the graph. Reconstruction of the reference sequence is shown. **d**, Example of a complex graph structure. Purple nodes form simple biallelic bubbles, while orange and blue nodes represent two branches of a superbubble (a bubble containing nested bubbles). **e**, Bubble chains formed from adjacent bubbles. Three bubble chains are shown; circles denote bubbles linked in a chain. The purple chain consists of two simple bubbles and a superbubble, while the nested blue and orange branches define separate bubble chains within the superbubble.

**Figure S2.** Flowchart of PangyPlot command line subcommands for setting up and running the application. During setup, the *pangyplot add* command ingests a GFA file, a primary reference path name, and two-dimensional coordinates generated by odgi layout. BubbleGun functionality was integrated into PangyPlot for identification of bubbles and bubble chains. GFA data is indexed into multiple SQLite databases. Gene annotations can be indexed with *pangyplot annotate*, which loads a GFF3 file into a separate database file. After setup, a Flask server can be launched with *pangyplot run* to respond to queries from the browser interface.

**Figure S3.** Interactive bubble popping reveals detail to the segment level. Top left: PangyPlot initially displays top-level bubbles, which are not nested within a higher order bubble (bubble chain in orange, individual bubbles in yellow). When a bubble is "popped" (denoted by dotted circle) the internal structure is revealed. Bubbles can be nested and repeated popping progressively exposes finer levels of variation until the graph resolves to individual segments (blue).

**Figure S4.** Canvas rendered view versus D3 implementation model. PangyPlot draws a graph segment such that it occupies screen space in proportion to its sequence length and independent of initial layout coordinates. Each segment is modelled as a series of D3 nodes joined end to end as a polyline (right view), scaled to its base-pair length and drawn thickened so the segment reads as a single unit (left view). This design allows relative segment sizes to be visualized while enabling long segments to bend naturally under the force-directed layout. Below a threshold of 10 bp, the polyline is replaced with a point using a single D3 node (e.g., Segment 426, 1 bp). Both the threshold and the rate of subdivision are tunable parameters, so the number of nodes representing a given segment can be adjusted.

**Figure S5.** Comparison of odgi layout to PangyPlot force-directed layout engine. PangyPlot initializes each graph segment with two-dimensional coordinates computed by odgi layout (grey). These coordinates are subsequently refined in real time using a D3 force-directed physics model, which incorporates node repulsion, collision handling, and link-length optimization to refine the layout (black). Arrows indicate matching structures.

**Figure S6.** Full view of chromosome 7 graph in PangyPlot. PangyPlot constructs a skeleton polyline view (white) of the variation graph from odgi layout coordinates, which enables the full chromosome to be viewed as a static image, similar to odgi draw. Locations of the *CFTR* and *PRSS1* genes are marked with annotations. A misassembly error in the underlying assembly data can be detected as the large structure with *POM121C* and *FAM185A* appearing near the breakpoints.

**Figure S7.** Comparison of graph visualization tools for examining *CFTR* exon 11 (p.Phe508del), polyT variant, and the *PRSS1*-*PRSS2* region. **a**, Linear view of the *CFTR* gene on GRCh38. Targeted region for visualization is highlighted in blue (exon 10 and 11, chr7:117,548,064-117,560,063). **b**, PangyPlot visualization showing annotated *CFTR* exons (yellow). A single haplotype with (TG)₁₀T₉ and p.Phe508del in *cis* is highlighted via red links. **c**, Bandage visualization with exon 10 and 11 manually annotated. **d**, Sequence Tube Map displays haplotypes as colored lines traversing sequence segments represented by white boxes. Two representative haplotype paths are highlighted: the (TG)₁₀T₉ allele containing the common CF-causing p.Phe508del deletion (blue) and the (TG)₁₀T₇ allele without p.Phe508del (orange). The view was manually truncated to the region of interest and annotations added during post-processing. **e**, A pair of one-dimensional visualizations from *odgi viz*. A representative subset of samples was chosen; haplotypes are drawn as rows. PolyT (left plot) and p.Phe508del (right plot) alleles are shown. **f**, Linear view of the *PRSS1*-*PRSS2* region on GRCh38 alternative contig KI270803.1, with GRCh38 alignments shown (chr7:142,743,462-142,778,093). KI270803.1 represents the 5-copy allele, the main chr7 contig uses the 3-copy allele. Functional trypsinogen genes are annotated in blue, pseudogenes in grey. **g**, PangyPlot visualization depicting *PRSS1*-*PRSS2* region, on initial rendering with top-level bubbles only (top). Expanding ("popping") the large bubble reveals additional CNV copies. The "REF/ALT" color style marks the GRCh38 reference path in blue and alternative segments in orange. The path animation feature (inset) enables real-time viewing of a single path highlighted along the graph. **h**, Bandage visualization of *PRSS1*-*PRSS2* region. BLAST search feature was used to identify trypsinogen genes, text annotations added manually. **i**, The *odgi draw* rendering of *PRSS1*-*PRSS2* region, line thickness indicates path depth (allele frequency).

**Figure S8.** Sequence Tube Map visualization of a complex *CFTR* region. Sequence Tube Map displays haplotypes as colored lines traversing sequence segments represented by white boxes. The figure shows a region within *CFTR* showing reduced interpretability due to looping paths and visually entangled haplotype structures, highlighting the limitations of Sequence Tube Map in representing regions with complex variation.

**Figure S9.** Two-dimensional odgi visualization of *CFTR* exon 10 and 11. Two-dimensional layouts of variation graphs generated using odgi draw. The figure shows a region encompassing *CFTR* exons 10 and 11. The high path density results in a tightly packed visualization where small variants are obscured.

**Figure S10.** HiFi reads align poorly at the *PRSS1*-*PRSS2* locus due to reference bias. Alignments of PacBio HiFi data to the *PRSS1*-*PRSS2* locus on GRCh38 (3-copy allele) from three different individuals. Each figure includes (from top to bottom): a Hifiasm assembly aligned to GRCh38, a track showing variant calls from HiFi read alignment, coverage track and phased HiFi reads colored by haplotype (pale red and blue are phased, grey is unphased). Soft clipping appears as dense colored segments. **a**, 3-copy / 3-copy individual. Alignments are clean, phased correctly, and exhibit minimal reference bias. Variant calling and coverage are consistent with expected structure. **b**, 5-copy / 5-copy individual. Soft-clipping present, local coverage spikes, a dense cluster of spurious variant calls, and phasing inconsistencies. **c**, 5-copy / 5-copy individual. All reads spanning the insertion region show soft clipping, with highly inflated coverage and phasing errors. These alignment artifacts reflect strong reference bias when mapping to GRCh38. All three individuals had accurately assembled haplotype assemblies.

# **Supplementary Tables**

**Table S1.** Preprocessing benchmark for each chromosome in the HPRC pangenome graph (PGGB graph and Minigraph-Cactus clip graph GRCh38 reference). Results cover only the PangyPlot indexing step; upstream preparation of the input (topological sorting with odgi sort and 2D layout generation with odgi layout) was performed separately. Wall-clock time and physical memory observed during the PangyPlot indexing step, measured per-chromosome build. All runs were performed on a laptop with a 2.1 GHz Intel Core i5 CPU and 16 GB RAM.

|  | **PGGB Time (min)** | **PGGB Peak Memory (GB)** | **Minigraph-Cactus Time (min)** | **Minigraph-Cactus Peak Memory (GB)** |
| --- | --- | --- | --- | --- |
| chr1 | 22.7 | 12.73 | 10.4 | 7.24 |
| chr2 | 11.7 | 8.01 | 10.9 | 7.23 |
| chr3 | 11.1 | 7.33 | 8.3 | 5.99 |
| chr4 | 10.7 | 7.02 | 8.9 | 6.17 |
| chr5 | 9.7 | 6.69 | 8.6 | 5.99 |
| chr6 | 8.3 | 5.85 | 7.7 | 5.47 |
| chr7 | 9.7 | 6.31 | 7.6 | 5.53 |
| chr8 | 8.1 | 5.37 | 6.9 | 5.50 |
| chr9 | 18.4 | 12.16 | 7.5 | 4.43 |
| chr10 | 7.9 | 5.79 | 6.5 | 5.16 |
| chr11 | 8.1 | 6.07 | 5.8 | 4.59 |
| chr12 | 7.1 | 5.69 | 5.6 | 4.46 |
| chr13 | 7.9 | 5.43 | 4.3 | 3.36 |
| chr14 | 7.3 | 5.33 | 4.0 | 3.54 |
| chr15 | 9.8 | 6.77 | 3.8 | 3.12 |
| chr16 | 9.4 | 5.10 | 4.5 | 3.95 |
| chr17 | 5.4 | 3.86 | 3.8 | 3.06 |
| chr18 | 5.4 | 4.29 | 3.5 | 2.75 |
| chr19 | 5.4 | 4.06 | 3.4 | 2.69 |
| chr20 | 5.3 | 4.05 | 3.0 | 2.57 |
| chr21 | 5.1 | 3.96 | 2.2 | 1.82 |
| chr22 | 6.8 | 5.37 | 2.1 | 1.86 |
| chrX | 6.7 | 5.00 | 5.1 | 4.39 |
| chrY | 0.5 | 0.69 | 0.2 | 0.42 |

**Table S2.** Preprocessing benchmark for each chromosome of the unreleased HPRC Release 2 Minigraph-Cactus pangenome clip graph (GRCh38 reference). Release 2 contains roughly five times as many samples (464 haplotypes) as the Release 1 graphs benchmarked in Supplementary Table 1. Results cover only the PangyPlot indexing step; upstream preparation of the input (2D layout generation with odgi layout) was performed separately. To enable indexing in PangyPlot, the compressed GBZ format graphs were indexed directly using a separate process, so these runtimes are not directly comparable to the GFA-based benchmark. Reported values are per chromosome: segment and link count, indexing time, peak memory, and total disk space of the resulting index. All runs were performed on a laptop with a 2.1 GHz Intel Core i5 CPU and 16 GB RAM.

|  | **Segments (millions)** | **Links (millions)** | **Index Time (min)** | **Peak Memory (GB)** | **Index Size (GB)** |
| --- | --- | --- | --- | --- | --- |
| chr1 | 10.6 | 14.7 | 25.5 | 10.40 | 1.81 |
| chr2 | 10.8 | 15.1 | 29.9 | 9.33 | 1.83 |
| chr3 | 8.4 | 11.6 | 19.3 | 8.41 | 1.43 |
| chr4 | 9.0 | 12.4 | 21.8 | 8.79 | 1.51 |
| chr5 | 8.3 | 11.4 | 18.1 | 8.67 | 1.39 |
| chr6 | 7.6 | 10.4 | 15.7 | 8.53 | 1.28 |
| chr7 | 7.7 | 10.7 | 16.5 | 7.94 | 1.30 |
| chr8 | 7.0 | 9.6 | 14.2 | 7.88 | 1.17 |
| chr9 | 5.9 | 8.2 | 11.7 | 7.35 | 1.01 |
| chr10 | 6.2 | 8.6 | 15.4 | 6.60 | 1.05 |
| chr11 | 5.9 | 8.2 | 11.3 | 7.31 | 1.00 |
| chr12 | 5.8 | 8.0 | 13.8 | 6.71 | 0.98 |
| chr13 | 4.3 | 6.0 | 8.1 | 5.29 | 0.73 |
| chr14 | 4.2 | 5.8 | 7.8 | 5.42 | 0.71 |
| chr15 | 5.4 | 7.5 | 9.7 | 6.30 | 0.87 |
| chr16 | 4.4 | 6.1 | 8.2 | 5.54 | 0.74 |
| chr17 | 3.9 | 5.4 | 7.0 | 5.38 | 0.65 |
| chr18 | 3.5 | 4.9 | 6.4 | 4.88 | 0.59 |
| chr19 | 3.5 | 4.9 | 6.2 | 4.52 | 0.58 |
| chr20 | 3.1 | 4.3 | 5.7 | 4.27 | 0.52 |
| chr21 | 2.0 | 2.7 | 3.6 | 2.74 | 0.33 |
| chr22 | 2.2 | 3.0 | 3.9 | 2.98 | 0.36 |
| chrX | 5.1 | 7.1 | 9.4 | 6.52 | 0.87 |
| chrY | 1.0 | 1.4 | 1.5 | 1.51 | 0.17 |

**Table S3.** Comparison of commonly used pangenome visualization tools. Comparison of implementation, strengths and weaknesses of Sequence Tube Map, odgi (viz and draw subcommands), Bandage, and PangyPlot.

|  | **Sequence Tube Map** | **odgi viz (1D)** | **odgi draw (2D)** | **Bandage** | **PangyPlot** |
| --- | --- | --- | --- | --- | --- |
| **Required Format** | vg | odgi | odgi | GFA | GFA |
| **Style** | Path-based, intersecting lines | Path-based, linear layout | 2D static image | Node-and-edge | Polyline skeleton + node-and-edge |
| **Interactive** | Yes | No | No | Yes | Yes |
| **File Size Limitations** | 5 MB | No specific limit | No specific limit | Must fit in memory | No specific limit |
| **Initial Layout** | Real-time calculation | Based on graph sorting | Precomputed via odgi layout | Real-time calculation | Precomputed via odgi layout |
| **Subsetting Required** | Yes | No | Optional | Yes for large graphs | No |
| **Implementation** | Browser-based | Command line | Command line | Desktop application | Browser-based |
| **Annotations** | None | Possible to inject into graph | Possible to inject via BED file | Requires external BED file | Built-in gene annotations |
| **Query Options** | Path coordinate-based | Range can be specified | - | Path coordinate, node ID, BLAST | Linear genome coordinates, gene-based |
| **Setup** | Install and deploy web app | Requires graph sorting | Requires graph sorting and layout computation | Minimal (load GFA in desktop app) | Layout computation, SQLite setup, app deployment |
| **Small Variant** | Yes | Not ideal | No | Yes | Yes |
| **Structural Variant** | Not ideal | Yes | Yes | Yes | Yes |
| **Chromosome-scale** | No | Yes | Yes | No | Yes |
| **Strengths** | Visualizing haplotype distribution | Visualizing haplotype distribution, structural variants | Visualizing large-scale graph structure | Rich features, low setup overhead | Pangenome scalable, built-in annotations, physics engine |
| **Challenges** | Subsetting, locating regions of interest, complex variants | Highly dependent of sorting method | Tuning of drawing parameters to match graph scale | Memory limitation, manual layout optimization | Initial data preprocessing and indexing |

**Table S4.** Features available in PangyPlot. The table enumerates 30 user-facing capabilities currently available in PangyPlot, grouped by category.

| **Category** | **Feature** | **Description** |
| --- | --- | --- |
| Navigation | Whole-chromosome view | Load and render an entire chromosome |
| Navigation | Multi-scale zoom | Zoom continuously from a chromosome overview to single-nucleotide resolution |
| Navigation | Reference-coordinate navigation | Navigate to a chr:start-end interval using linear reference coordinates |
| Navigation | Live coordinate readout | Display the linear coordinates for the visible subgraph range |
| Navigation | Cytoband ideogram | Select a chromosome and drag to choose a region from an ideogram |
| Navigation | Gene search | Locate a gene by name and navigate to it |
| Navigation | Shareable view URLs | Encode the displayed region in the URL for sharing and revisiting |
| Graph exploration | Bubble expansion | Expand a bubble to reveal its internal segments, iteratively to the level of individual bases |
| Graph exploration | Undo expansion | Reverse the most recent bubble expansion |
| Graph exploration | Chain view | Present variation as bubble chains that act as single units |
| Graph exploration | Force-directed arrangement | Arrange graph elements automatically in real time |
| Graph exploration | Chain manipulation | Drag a chain to reposition it or flip its orientation |
| Graph exploration | Element inspection | Report identifier, coordinates, length, GC content, sequence and nesting for any element |
| Annotation | Gene annotation display | Overlay gene features on the graph |
| Annotation | Annotation management | Show, hide, recolor, star or filter individual genes; display exons only |
| Annotation | Custom annotations | Define and label user-specified regions of the graph |
| Haplotype paths | Haplotype tracing | Highlight the path of a selected sample through the displayed region |
| Haplotype paths | Path animation | Animate traversal of a haplotype with playback controls |
| Export | Subgraph export | Export a selected subgraph in GFA format |
| Export | Layout export | Export a selected subgraph layout coordinates in odgi and Bandage formats |
| Export | Image export | Save the current view as a PNG or SVG image |
| Export | Coordinate export | Copy the coordinates of the current selection |
| Display | Color schemes | Color elements by node type, bubble size, length, reference/alternate status, position or GC content |
| Display | Color presets | Apply predefined palettes, including colorblind-friendly options |
| Display | Layout controls | Adjust force-simulation parameters interactively |
| Data preparation | GFA ingestion | Index a GFA file against a chosen reference path; P-line and W-line formats are supported |
| Data preparation | Annotation ingestion | Load gene annotations from a GFF3 file |
| Data preparation | Non-model organism support | Generate a pseudo-ideogram from a FASTA index for organisms without cytoband data |
| Data preparation | Containerized deployment | Deploy from a prebuilt container without manual dependency installation |
| Data preparation | Multi-language interface | Present the interface in eleven languages |

## Supplementary Methods

## Supplementary Methods 1

**Data storage and querying.** PangyPlot uses SQLite tables for durable record storage of the graph, holding the complete record of every graph element. SQLite was chosen because it is portable, server-less, quick to add rows, and modest with memory use. Four databases are built per chromosome. 1) The segment table holds one row per GFA S line, recording the sequence, its length, GC and ambiguous base counts, and the two-dimensional layout coordinates. 2) The link table holds one row per L line, recording the two segment ends joined by the link and the fraction of haplotypes traversing it. 3) The step table holds one row per step along the reference path and includes step index and reference position. 4) The bubble table holds one row per bubble, recording its source, sink, interior segments, and nesting hierarchy. A fifth database, built separately when gene annotations are loaded, holds one row per GFF3 feature. To increase query speed, PangyPlot complements the SQLite backend with compact in-memory indexes built at startup from precomputed caches. For example, lightweight per-segment attributes such as length and layout coordinates are held in parallel typed arrays in memory sorted by segment ID, allowing constant-time lookup without a database query. SQLite is queried only when the full record of a specific element is required.

Not all data is held in SQLite. Haplotype paths are stored as one delta-encoded binary file per path and streamed to the client. The chromosome-scale skeleton representation is a set of precomputed simplified polylines stored in static compressed files; the bubble chains are stored similarly. These structures are read in bulk rather than searched, so a database offers no benefit over a flat file.

## Supplementary Methods 2

**Construction of variation graph with Minigraph-Cactus.** We employed the Minigraph-Cactus pangenome pipeline v2.7.2, to construct variation graphs from de novo assemblies [1]. Minigraph-Cactus was chosen due to its scalability with larger sample sizes. We constructed a variation graph using chromosome 7 scaffolds from 101 individuals with cystic fibrosis (hybrid assemblies of PacBio continuous long reads and 10x Genomics linked reads) and reference sample HG002. To assign de novo scaffolds to human chromosomes, we aligned each assembly to CHM13 using minimap2 2.24 with the --asm5 flag and output in PAF format [2]. Non-aligning scaffold tips were clipped from the assembly. Chromosome 7 sequence from CHM13 and GRCh38 (chr7 + nine alternative contigs) were also included in the graph.

## Data and Code Availability

A server with preloaded HPRC data is available at https://pangyplot.research.sickkids.ca. PangyPlot source code is available at https://github.com/strug-hub/pangyplot under the MIT License. The version described here (v0.3.0) is archived at https://doi.org/10.5281/zenodo.21420030. A prebuilt container image bundling all dependencies is available at https://github.com/strug-hub/pangyplot/pkgs/container/pangyplot. Detailed usage instructions, API documentation, and example datasets are provided at https://pangyplot.readthedocs.io/. HPRC Minigraph-Cactus GFA and layout files: https://doi.org/10.5281/zenodo.15311998 HPRC PGGB GFA and layout files: https://doi.org/10.5281/zenodo.19580038 Indexes for HPRC Minigraph-Cactus (hosted on server): https://doi.org/10.5281/zenodo.17174108

## Acknowledgements

CGMS was approved by the Research Ethics Board of the Hospital for Sick Children (#0020020214 from 2002 to 2019 and #1000065760 from 2019 to the present) and all participating sub-sites. Written informed consent was obtained from all participants or parents/guardians/substitute decision makers prior to inclusion in the study. We thank the patients, care providers, clinic research assistants, collaborators, and principal investigators at CF Centers across Canada for their contributions to the Canadian CF Gene Modifier Study (https://github.com/strug-hub/Canadian-Cystic-Fibrosis-Gene-Modifier-Study), CF Canada Patient Registry and the CF Canada-SickKids Program in Individual Therapy. PangyPlot builds upon existing graph algorithms, notably the layout algorithm from odgi and the bubble detection algorithm from BubbleGun. PangyPlot was also bootstrapped by the D3 force graph implementation by Vasco Asturiano (https://github.com/vasturiano/force-graph). We thank the developers for making these tools available. We thank Laurie Lintott for helpful comments on the manuscript draft.

## Canadian Cystic Fibrosis Gene Modifier Consortium

Lisa J Strug¹, Katherine Keenan¹, Felix Ratjen¹, Johanna Rommens¹, Melinda Solomon¹, Candice Bjornson², Mark Chilvers³, April Price⁴, Michael Parkins⁵, Michael Derynck⁶, Emmanuelle Brochiero⁷, Lara Bilodeau⁸, Dimas Mateos-Corral⁹, Daniel Hughes⁹, Mary Jane Smith¹⁰, Nancy Morrison¹¹, Janna Brusky¹², Anne Stephenson¹³, Elizabeth Tullis¹³, Bradey Quon¹⁴, Jaled Yehya¹⁵, Winnie M Leung¹⁵, Andre Cantin¹⁶, Larry Lands¹⁷

1. The Hospital for Sick Children, Toronto, ON, Canada. 2. Alberta Children's Hospital, Calgary, AB, Canada. 3. BC Children's Hospital, Vancouver, BC, Canada. 4. Children's Hospital of Western Ontario (London Health Sciences Centre), London, ON, Canada. 5. Foothills Medical Centre, Calgary, AB, Canada. 6. Kingston Health Sciences Centre, Kingston, ON, Canada. 7. Centre de recherche du Centre hospitalier de l'Université de Montréal (CRCHUM), Montréal, QC, Canada. 8. Institut universitaire de cardiologie et de pneumologie de Québec (IUCPQ), Québec, QC, Canada. 9. IWK Health Centre, Halifax, NS, Canada. 10. Janeway Children's Health & Rehabilitation Centre, St. John's, NL, Canada. 11. Queen Elizabeth II Health Sciences Centre, Halifax, NS, Canada. 12. Royal University Hospital, Saskatoon, SK, Canada. 13. St. Michael's Hospital, Toronto, ON, Canada. 14. St. Paul's Hospital, Vancouver, BC, Canada. 15. University of Alberta Hospital, Edmonton, AB, Canada. 16. Centre hospitalier universitaire de Sherbrooke (CHUS), Sherbrooke, QC, Canada. 17. Montreal Children's Hospital, Montréal, QC, Canada.

# Supplementary References

1. Hickey et al., 2024, Pangenome graph construction from genome alignments with Minigraph-Cactus

2. Li et al., 2018, Minimap2: pairwise alignment for nucleotide sequences
